# Association of osteoarthritis and circulating adiponectin levels: a systematic review and meta-analysis

**DOI:** 10.1186/s12944-018-0838-x

**Published:** 2018-08-16

**Authors:** Qian Tang, Zhi-Chao Hu, Li-Yan Shen, Ping Shang, Hua-Zi Xu, Hai-Xiao Liu

**Affiliations:** 1 Department of Orthopaedic Surgery, The Second Affiliated Hospital and Yuying Children’s Hospital of Wenzhou Medical University, 109, Xueyuanxi road, Wenzhou, 325027 China; 2 Department of Rehabilitation, The Second Affiliated Hospital and Yuying Children’s Hospital of Wenzhou Medical University, 109, Xueyuanxi road, Wenzhou, 325027 China

**Keywords:** Osteoarthritis, Adiponectin, Meta-analysis

## Abstract

**Background:**

The objective of this study was to perform a meta-analysis to investigate the specific relationship between the expression level of circulating adiponectin and osteoarthritis (OA).

**Method:**

Multiple databases were searched to estimate the high quality of studies relevant to adiponectin and OA. We extracted the data from the eligible studies and included them in the meta-analysis using a random effects model. Subgroup analysis and meta-regression were further performed to explore the potential sources of heterogeneity.

**Results:**

Ten articles consisting of thirteen case-control studies that contained a combined total of 1255 subjects. Our results revealed that the OA patients displayed higher adiponectin levels than the healthy controls (SMD = 0.327, 95% CI: 0.11–0.55, *P* = 0.003). The ethnicity-stratified subgroup analysis indicated that the adiponectin was a sensitive biomarker in both Caucasians (*P* = 0.021) and Asians (*P* = 0.037). Moreover, the meta-regression analysis suggested that the sample size (*P* = 0.03) and nationality (*p* = 0.01) could account for a part of heterogeneity in our study.

**Conclusion:**

Taken together, the current study indicated that the adiponectin expression levels were higher in the OA patients than in the healthy controls and might be associated with OA prevalence.

## Background

Osteoarthritis (OA), a painful degenerative joint disease involving articular cartilage loss, subchondral bone remodeling, osteophyte formation and soft tissue damage, causes ongoing disability in elderly people due to its irreversible outcome [1, 2]. The worldwide incidences of knee and hip OA are estimated to be 6–17% and 2–10%, respectively and the condition especially affects women over 60 years of age [3]. It has become the main reason for total joint arthroplasty (TJA) and is therefore to the source of a considerable economic burden [4]. While the etiology of osteoarthritis [OA] is still not clearly understood [5, 6], the evidence suggested that OA is a systemic disorder with a multifactorial origin. The systemic risk factors include obesity, gender, injury, age and a genetic bias [2]. Moreover, there are increasing evidences indicated that obesity is an essential element in the pathogenesis of OA [7]. Sowers et al. [8] have suggested that the mechanism by which obesity is involved in OA, may be a simple increase under the mechanical burden in the joints. However, Gabay et al. [9] indicated that the obesity induced high metabolic and inflammatory environments play crucial roles in the onset of OA. What’s more, several early studies have indicated that there is an association between OA and some adipokines in serum or synovial fluid [10–13]. One of these mediators of interest is adiponectin, which has been shown to be in relation to OA [14].

Adiponectin, a 28–30 kDa collagen-like protein, not only is one of the most abundantly secreted adipose tissue proteins but also is the only adipokine identified thus far that is negatively correlated with obesity [15]. For many years, the effects of adiponectin in many metabolic conditions such as insulin resistance, atherosclerosis, and myocardial infarction have been intensely studied [16–18], as well as its roles in inflammatory and anti-inflammatory processes [19]. However, the effects of adiponectin in OA process were still controversial. On the one hand, evidence had shown that both human and murine chondrocytes express functional adiponectin receptors (AdipoR1 and AdipoR2) [20]. In cultured chondrocytes, adiponectin treatment leads to a dose-dependent increase of the pro-inflammatory factors such as inducible nitric oxide synthase (iNOS), interleukin-6 (IL-6) and metalloproteases (MMPs) [21, 22]. All of those factors may degrade matrix, destroy articular cartilage and eventually result in OA [23]. What’s more, several studies supported that the higher level of adiponectin was presented in both the plasma and synovial fluid of OA patients compared with the healthy controls [14, 24–29]. In addition, a recent study found there was a positive correlation between adiponectin concentration and the Kellgren-Lawrence (KL) grading scores (A scoring system describes the degree of joint degeneration via X-ray image system) [14]. On the other hand, a few studies have failed to demonstrate a statistical association between adiponectin and OA [20, 30, 31]. Moreover, some data suggest that the adiponectin expression level is negatively correlated with the radiographic severity of OA and might be playing a protective role in the pathogenesis of OA [32, 33]. To examine what role the adiponectin plays in OA process, we conducted this meta-analysis to evaluate the relationship between the expression level of adiponectin and OA prevalence.

## Methods

### Searching strategy

**Fig. 1:**
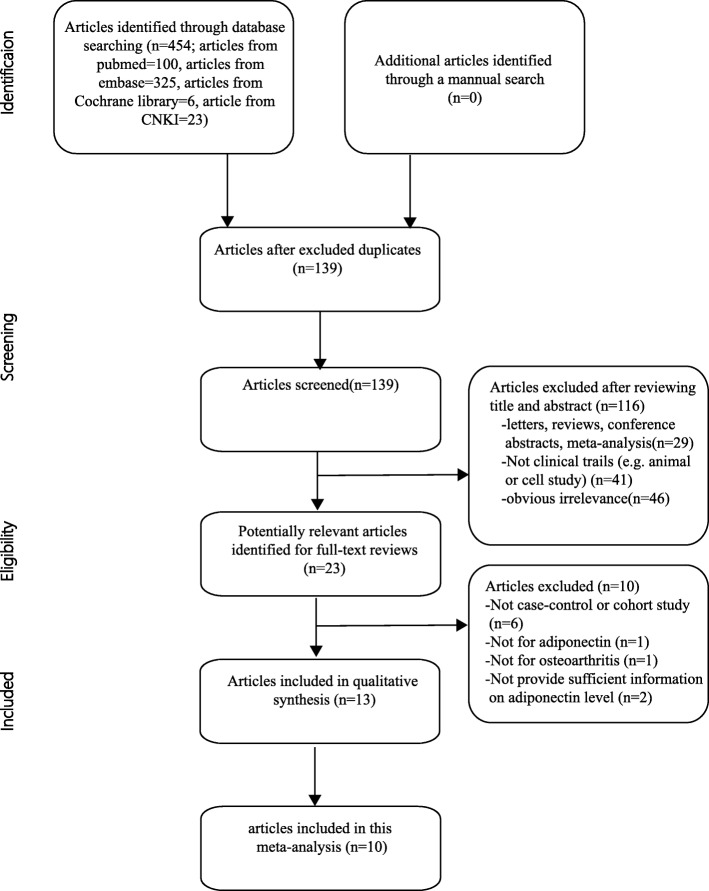
Flow chart of the articles selection and inclusion process

In this meta-analysis, we followed the guidelines regarding the preferred reporting items recommended in the systematic reviews and meta-analyses (PRISMA) statement [34] to identify the relevant studies written in English or non-English languages, which were found by searching the following electronic databases: MEDLINE, Embase, Cochrane Library, China National Knowledge Infrastructure (CNKI), and Google Scholar. In addition, further relevant studies including those shown in the reference lists of all the included studies were also manually searched. The last search was carried out on August 10, 2017. The following search terms (“Osteoarthritis, Knee” or “Osteoarthritis, hip” or “Osteoarthritis, spine” or “Osteoarthritis” or “knee osteoarthritis” or “hip osteoarthritis” or “spine osteoarthritis” or “spinal osteoarthritis” or “lumbar osteoarthritis” or “hand osteoarthritis”) and (“Adiponectin” or “adiponectin” or “Obese Protein” or “Obese Gene Product”) were used. The search strategy is presented in Fig. 1.

### Selection criteria

Studies meeting the following criteria were included: 1) the basic requirement was that the studies were restricted to human populations and published in a peer-reviewed journal; 2) only case-control or cohort studies that measured the expression levels of adiponectin in both osteoarthritis patients and healthy controls were included; 3) a minimum sample size of least ten was required; 4) all of the OA patients were identified and confirmed by the Diagnostic and Therapeutic Criteria Committee of the American Rheumatism Association [35]; 5) the original data and sufficient information were provided regarding adiponectin serum or synovial levels in the OA patients and healthy groups. The major exclusion criteria were as follows: 1) the studies did not satisfy the above requirements; 2) the studies included duplicate publications, for example, studies published by the same author or having data derived from the same clinical trial.

### Data extraction

For each eligible study, two of the authors of the present study independently extracted all of the relevant data following the criteria above, and disagreements were resolved by discussion with a third investigator. For each publication, the following variables were abstracted: the surname of the first author, publication year, source of publication, study design, sample size, patient genders, mean age, Body Mass Index (BMI), country, ethnicity, joint involvement, stage of OA, expression level of adiponectin, source of the samples from the controls and subjects used for the adiponectin analysis and the detection method. To evaluate the effects of the various ethnicities of the subjects, we collected this information separately and classified the subjects into Asians and Caucasians. And the subgroup evaluation was also conducted by dividing parts into two groups according to the KL score or radiological findings: early OA group (KL score = 1 or without radiological change) and middle and late OA group (KL score = 2,3,4 or with radiological change). All authors approved the final inclusion of studies for meta-analysis.

### Quality assessment

To ensure a high quality assessment of the included studies, two experienced reviewers independently applied the Newcastle–Ottawa scale (NOS) [36] Using this tool, each study was judged on eight items, categorized into the following three groups: 1) the selection of the study groups; 2) the comparability of the groups; 3) the assessment of either the exposure or the outcome of interest for case–control or cohort studies, respectively. Stars were awarded for each quality item to serve as a quick visual assessment. Up to nine stars were awarded to the highest quality studies. A third reviewer was consulted if there had been any disagreement between the two investigators regarding the any of the elements of NOS scores.

### Statistical analysis

This meta-analysis was undertaken using STATA 12.0 software (Stata Corp, College Station, TX, USA). A standardized mean difference (SMD) for the adiponectin expression levels and the 95% CIs were calculated for the generic inverse variance outcomes between the cases and controls in accordance with the Z-test. According to the statistical heterogeneity was considered to be present at *P* < 0.05 and the I^2^ values were > 50% in our study, a random effects model was applied and further subgroup study and meta-regression analysis were performed to detect the origin of heterogeneity. To test the strength and stability of the pooled results, we performed a sensitivity analysis by omitting the individual studies one by one. Moreover, the effect of publication bias was investigated by egger’s test.

## Results

### Literature search outcome

The search strategy (Fig. 1) identified 454 relevant studies from the databases listed above. After reviewing the titles, abstracts and the full-text of the articles, only ten articles consisting of thirteen case-control studies, which included 754 OA patients and 501 healthy controls, remained for our meta-analysis. The quality scores for the methods used in the thirteen included studies are presented in the end of Table 1. Information on the joint involvement, subjects’ ethnicity, the stage of OA and source of samples was provided in all of these studies. The BMI scales were provided in eleven studies. Table 1 summarizes the basic characteristics and the information provided in the included studies. The scores for the quality of the methodology of the ten included studies are also presented in Table 1.

**Table 1:** Characteristics of the included studies

Reference	Year	Ethnicity	Country	Position	Sample Size		Female ratio(%)		Mean age (years)		Mean BMI(kg/m^2^)		Method	NOS
					Case	Control	Case	Control	Case	Control	Case	Control		
Senolt [29]	2006	Caucasians	Czech Republic	Knee	21	23	73.9	73.9	66.15	58.13	27.1	25.5	ELISA	7
Filkova(a) [30]	2009	Caucasians	Czech Republic	Hand	27	20	100.0	100.0	NA	NA	NA	NA	ELISA	6
Filkova(b) [30]	2009	Caucasians	Czech Republic	Hand	48	20	100.0	100.0	NA	NA	NA	NA	ELISA	6
Tan [21]	2009	Asians	China	Knee	35	20	85.7	85.0	50.1	45.9	19.3	20.1	ELISA	7
Honsawek [22]	2010	Asians	Thailand	Knee	76	24	81.6	79.2	69.8	71.2	26.1	25.5	ELISA	7
De Boer [31]	2012	Caucasians	Netherlands	Knee	172	132	69.2	74.2	67.4	56.5	29.5	28.1	ELISA	8
Choe(a) [23]	2012	Asians	Korea	Hand	50	46	NA	NA	61.6	57.6	24.1	23.9	ELISA	8
Choe(b) [23]	2012	Asians	Korea	Hand	60	46	NA	NA	63.5	57.6	24.1	23.9	ELISA	8
Wang [32]	2014	Asians	China	Knee	64	19	73.4	73.6	62.4	39.1	26.1	25.5	ELISA	8
Liu [33]	2015	Asians	China	Knee	71	31	31.0	35.5	46.7	44.9	26.1	25.9	ELISA	8
Cuzdan Coskun(a] [14]	2015	Caucasians	Turkey	Knee	30	25	90.0	52.0	67.0	43.1	23.4	27.5	ELISA	6
Cuzdan Coskun(b) [14]	2015	Caucasians	Turkey	Knee	30	25	100..0	52.0	64.5	43.1	33.6	27.5	ELISA	6
Tootsi [34]	2016	Caucasians	Estonia	Knee and Hip	70	70	50.0	48.6	62.0	60.0	28.0	26.0	ELISA	7

*M* Male, *F* Female, *ELISA* Enzyme-linked immunosorbent assay, *NA* Not available

### Study characteristics

**Fig. 2:**
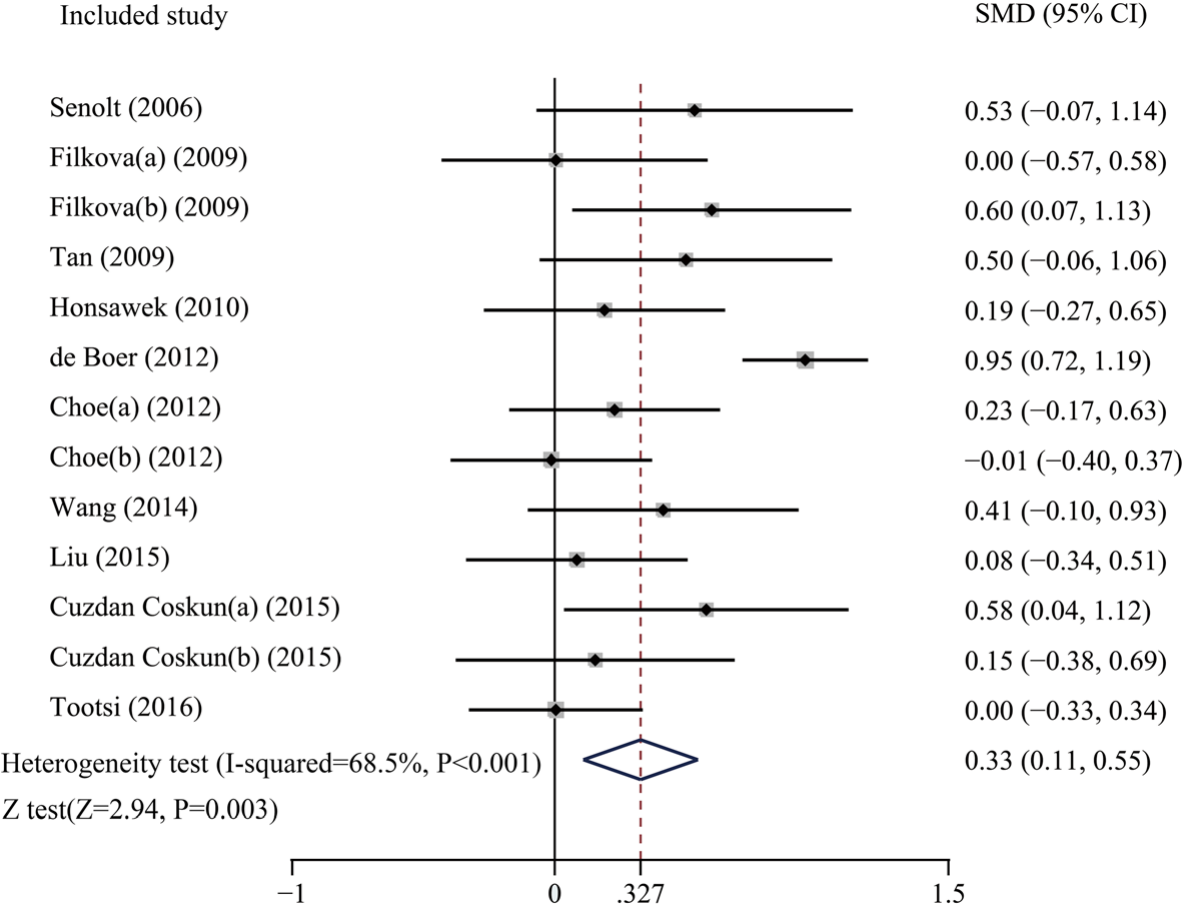
Forest plot for the clinical significance of adiponectin expression level in osteoarthritis patients compared with healthy controls. SMD, standardized mean difference; Cl. confidence interval

**Fig. 3:**
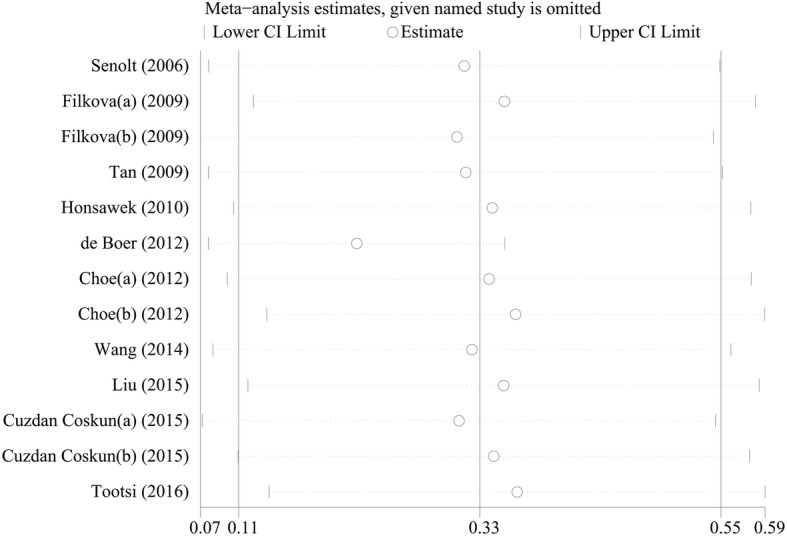
Sensitivity analysis was performed through omitting individual studies one by one to evaluate whether the overall results could have been significantly influenced by one single study

**Fig. 4:**
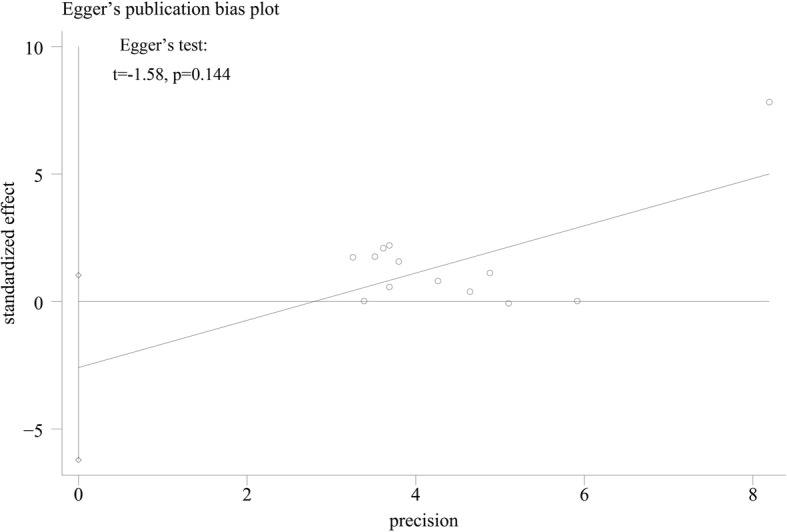
Egger’s plot was performed to evaluate the publication bias of included studies

The pooled results indicated that the expression level of adiponectin was significantly higher in the OA patients compared with that in the healthy groups (SMD = 0.327, 95% CI: 0.11–0.55, *P* = 0.003, Fig. 2). The causes of heterogeneity in the results were explored according to our a priori hypotheses by subgroup analysis and meta-regression. The results of subgroup analysis showed that the higher expression level of adiponectin was observed only in the knee OA patients, but was not significantly observed in the subjects with hand OA or knee and hip OA. (Knee OA: SMD = 0.44, 95% CI: 0.17–0.72, *P* = 0.002; Hand OA: SMD = 0.18, 95% CI: -0.07–0.44, *P* = 0.161; Knee and hip OA: SMD = 0.00, 95% CI: -0.33–0.34, *P* = 0.980, Table 2). Meanwhile, the results indicated that adiponectin might be a biomarker for OA in both Caucasian and Asian subjects as analyzed in current study (Caucasians: SMD = 0.42, 95% CI: 0.06–0.71, *P* = 0.021; Asians: SMD = 0.19, 95% CI: 0.01–0.18, *P* = 0.037, Table 2). What’s more, the stage-stratified analysis showed that the adiponectin expression levels were correlated with OA in the patient who entered the later stage of OA, but in the early OA patients group, we failed to get the similar results (early OA: SMD = 0.00, 95% CI: -0.57–0.58, *P* = 0.356; middle and late OA: SMD = 0.23, 95% CI: -0.17–0.63, *P* = 0.004, Table 2). In addition, the separate variable and multivariable meta-regression analyses for adiponectin expression level were performed to analyze the potential sources of inter-study heterogeneity. Overall, the sample size and the nationality of patients might be the major sources of heterogeneity for our study (Sample size: *p* = 0.03; Korea vs Czech Republic: *p* = 0.01, Table 3). Furthermore, the sensitivity analysis indicated that no significant differences resulted from the omission of the data from any single study (Fig. 3). and the Egger’s test showed that there is no publication bias in this meta-analysis (*t* = − 1.58, *P* = 0.144, Fig. 4).

**Table 2:** Subgroup analyses stratified by various factors

Factors	Subgroups	Studies (n)	Patients(n) (Case/Control)	I^2^(%)	Heterogeneity (p)	SMD	95% CI	Z test (p)
Ethnicity	Caucasians	7	398/315	77.7	< 0.001	0.42	0.06–0.71	0.021
	Asians	6	356/186	0.0	0.661	0.19	0.01–0.18	0.037
Joint involvement	Knee	8	499/299	65.7	0.005	0.44	0.17–0.72	0.002
	Hand	4	185/132	20.1	0.289	0.18	-0.07-0.44	0.161
	Knee and Hip	1	70/70	–	–	0.00	-0.33-0.34	0.980
Stage	early OA	2	77/66	0.0	0.533	0.00	-0.57-0.58	0.356
	Middle & late OA	11	677/435	71.8	< 0.001	0.23	-0.17-0.63	0.004

**Table 3:** Meta-regression of single variable and multivariable

Variables	Number of comparisons	β	95%Cl	P	R^2^
Single variable					
Publication Year	13	−0.03	−0.11-0.40	0.40	0.68
Sample size	13	0.0002	0.004–0.03	*0.03*	0.53
Country(vs Czech Republic)	13				0.99
China	3	0.99	−0.45-0.64	0.67	
Thailand	1	−0.28	−0.83-0.26	0.25	
Netherlands	1	−0.18	−0.68-0.31	0.40	
Korea	2	0.66	0.19–1.13	*0.01*	
Turkey	2	−0.099	−0.78-0.58	0.73	
Estonia	1	0.072	−0.52-0.66	0.77	
Location(vs knee)	13				0.16
Hand	2	0.21	−0.20-0.73	0.24	
Knee and Hip	1	0.34	−0.95-0.57	0.60	
Stage(secondary OA/primary OA)	13	0.22	−0.39-0.85	0.43	0.69
Multivariable					
Mean of Age(years)					0.02
OA	13	0.02	−0.02-0.06	0.28	
Control	13	−0.01	−0.04-0.01	0.40	
Gender(female%)					−0.09
OA	13	−0.002	−0.02-0.02	0.98	
Control	13	0.05	−0.01-0.02	0.52	
Mean of BMI					0.06
OA	13	−0.04	−0.16-0.08	0.43	
Control	13	0.10	−0.09-0.30	0.26	

A value of *P* < 0.05 was considered statistically significant

## Discussion

In this meta-analysis, the major finding was that expression level of adiponectin was significantly higher in the OA patients than in healthy subjects. This result might support those of a previous study that indicated that the metabolism of adipose tissue was a key factor that contributed to the OA development [37] and the adiponectin might be closely related to the pathogenesis of OA.

However, with respect to the molecular mechanism, it is still a matter of dispute whether adiponectin plays a protective role or an effector role. Some investigators considered its function to be primarily a pro-inflammatory mediator that may cause OA [10], whereas others hold the opposite opinion [20]. In clinical studies, the relationship between the adiponectin levels and the severity of the OA is also controversial. Most investigators have suggested that adiponectin plays a protective role in OA on the basis of its negative correlation with disease progression [30, 31], and all of patients included in above studies was with middle and late OA. Interestingly, however, our pooled analyses also suggest that adiponectin was significantly up-regulated in middle and late stage of OA compared with healthy control. The expression of adiponectin might therefore be a pivotal biomarker that could help to the diagnosis of the progression of OA. Moreover, a study reported by StuM Otero et al. held the opinion that the increased levels of adiponectin in patients with arthritis suggested a compensatory mechanism under catabolic or anabolic imbalance [38]. Meanwhile, Aspden et al. in their study suggested that osteoarthritis is a systemic disorder in which promote lipid metabolism with its progression [39]. However, the circulating adiponectin level is natively associated with level of lipid metabolism [40]. Thus, we hypothesized that when the disease progressed, the adiponectin levels may be down-regulated in response to the elevating level of lipid metabolism. It would provide a new therapy target for OA if our hypothesis be confirmed in the future.

The results of our subgroup analysis also revealed that this association was stronger in the patients with knee OA but might not with hand OA or knee and hip OA. Bias might be present for the knee and hip OA group as there was only one study in the literature. Therefore, the clinical applicability of our conclusion should be taken into consideration with other major diagnostic strategies in hand or hip OA patient. As for knee joint, one of weight-bearing joint most influenced by obesity, was confirmed to be more susceptibility through the action of inflammatory adipokines than hip joint [41]. This finding was consistent with our result. However, Arita et al. [15] discovered the adiponectin in obese subjects were significantly lower than that in non-obese one, although adiponectin was secreted only from adipose tissue. Which suggested a more complex relationship among obesity, adiponectin and OA and might further approve of our hypothesis that adiponectin increase compensatorily in the OA patient. Furthermore, the ethnicity-stratified analysis showed that the adiponectin expression level was higher in both Caucasian and Asian OA subjects than in the controls, which indicated ethnicity differences may not substantially affect the outcomes. In addition, we cannot get sufficient information about gender difference in this issue owing to result of meta-regression analyses, although lots of researchers such as Perruccio et al. [42] have found that adiponectin level was higher in female than male, suggesting the adiponectin might be a sensitive predictor in women with OA.

However, it is worth mentioning that the significant heterogeneity was detected when we combined the SMD. Because the varied characteristics of all included studies might have influenced the heterogeneity such as sample size, study designs, patient characteristics. Therefore, we used a random-effect model to synthesize the data on the basis of large population. The subgroup study and meta-regression analyses were performed to detected the potential sources of heterogeneity. In the result, we found that the sample size and nationality of patients might explain a part of heterogeneity, which suggested some high quality studies consisting of enough sample and varies of countries were needed to this issue in the future. Furthermore, the result of sensitive analyses and egger test were also supporting the stability of our conclusion.

Excepting heterogeneity, the current meta-analysis has several limitations. First, to improve the level of evidence, we restricted the inclusion criteria of included studies which must contained control group so that several cross-sectional studies were excluded. The data were therefore inadequate to estimate the potential relationship between the severity of the OA and the adiponectin levels. Therefore, based on our study, a further meta-analysis that will include all of the related observational studies will be necessary to evaluate the specificity of the association between adiponectin and the severity of OA. Second, in the ethnicity-stratified analysis, we did not have any information regarding mixed populations or black people, so the conclusion may not represent the worldwide distribution of ethnicities. Third, several unpublished papers and meeting abstracts were not taken into account because the data required for the inclusion and exclusion criteria was unavailable, which may cause a potential selection bias. In spite of these limitations, our study is the first meta-analysis to investigate the correlation between the adiponectin expression level and OA prevalence.

In conclusion, our meta-analysis revealed that the adiponectin expression level was up-regulated in later stage of OA especially in knee OA patients. Most importantly, these results may therefore provide a potential reliable tool for synergistically diagnosing OA, demonstrating its pivotal clinical significance and might be a potential target for the OA therapy. Further research with standardized, unbiased methods and larger sample sizes are required for deeper analysis.
